# Promoting remyelination in multiple sclerosis

**DOI:** 10.1007/s00415-019-09421-x

**Published:** 2019-06-12

**Authors:** Nick Cunniffe, Alasdair Coles

**Affiliations:** Department of Clinical Neurosciences, University of Cambridge, Cambridge, UK

**Keywords:** Multiple sclerosis, Remyelination, Clinical trials, Visual evoked potentials, Magnetisation transfer ratio

## Abstract

The greatest unmet need in multiple sclerosis (MS) are treatments that delay, prevent or reverse progression. One of the most tractable strategies to achieve this is to therapeutically enhance endogenous remyelination; doing so restores nerve conduction and prevents neurodegeneration. The biology of remyelination—centred on the activation, migration, proliferation and differentiation of oligodendrocyte progenitors—has been increasingly clearly defined and druggable targets have now been identified in preclinical work leading to early phase clinical trials. With some phase 2 studies reporting efficacy, the prospect of licensed remyelinating treatments in MS looks increasingly likely. However, there remain many unanswered questions and recent research has revealed a further dimension of complexity to this process that has refined our view of the barriers to remyelination in humans. In this review, we describe the process of remyelination, why this fails in MS, and the latest research that has given new insights into this process. We also discuss the translation of this research into clinical trials, highlighting the treatments that have been tested to date, and the different methods of detecting remyelination in people.

## Introduction

Multiple sclerosis (MS) is a chronic, primarily inflammatory, disorder of the central nervous system (CNS) characterised by focal lymphocytic infiltration causing damage to myelin and axons [1-3]. Typical clinical features include weakness, sensory loss, diplopia, reduced visual acuity, dysarthria, dysphagia, ataxia, and bladder dysfunction; largely a reflection of the distribution of demyelinating foci throughout the CNS. In 85% of patients, there is an initial period of episodic neurological dysfunction followed by partial or complete recovery (relapsing–remitting MS, RRMS) [4]. Over time, the clinical picture often develops to one of progressive disability (secondary progressive MS, SPMS) [5], while in 15% the illness is progressive from the outset (primary progressive MS, PPMS) [6, 7]. In both SPMS and PPMS, the strongest predictor of the onset of progression is age, typically being seen from around 40 years [8].

There now exists an extensive therapeutic armamentarium against the inflammation of MS [9]. These disease-modifying treatments (DMTs) reduce the incidence and severity of new lesions by limiting the activity and availability of immune cells, manifesting clinically through reductions in relapse rates and disability accrual [10-19]. However, the “therapeutic window” for treatment with these immunotherapies is limited [20]; best long-term results on disability are seen if an anti-inflammatory treatment is started within 5 years of the first clinical episode of demyelination in relapsing–remitting disease [21]. Furthermore, while the 16 current DMTs are licensed for RRMS, only one—ocrelizumab—is approved for primary progressive disease [22], and even then its effects are so modest that several reimbursement agencies, notably NICE in the UK, declared it only cost-effective in a subset of people with new or contrast-enhancing lesions on MRI and a disease duration of less than 15 years.

Instead, the promotion of regeneration of the myelin sheath, through enhancing the process of endogenous remyelination, has emerged as one of the most amenable prospects to delay, prevent or reverse progression [23]. This is grounded in experimental evidence that demonstrates that the myelin sheath (and its associated oligodendrocytes) does not just facilitate nerve conduction, but is also directly protective against degeneration [24-29]. In this review, we describe the biology of remyelination, why this fails in MS, and recent research that has raised questions about current approaches to promoting remyelination. We then consider the therapies that have been, and are being, evaluated and discuss the best approach to measure remyelination in people, which is proving increasingly essential as we translate promising preclinical research into clinical trials.

## The role of myelin

Myelination offers a far better way of increasing the conduction velocity of nerve fibres than simply increasing axon size. Myelin increases the transverse, insulating, resistance of the axon membrane, while the voltage-gated sodium and potassium channels are virtually confined to short unmyelinated nodes of Ranvier. The action potential is therefore propagated by the comparatively rapid and energy-efficient process of saltatory conduction [30, 31]. It follows that loss of myelin leads to slower transmission of the action potential, and hence prolonged latency, but can also lead to conduction block [32]. Remyelination is therefore a way to restore saltatory conduction [33], and clinical function [34], after demyelination.

Additionally, oligodendrocytes directly support the neuron, for example through providing lactate for metabolism and generation of ATP [25-27]. Pathological studies and animal models also suggest that axonal degeneration is reduced in remyelinated areas [24, 28, 29]. Taken together, the rationale for a remyelinating therapy is to restore function and prevent neurodegeneration.

Meanwhile, it is increasingly apparent that myelin regulation is a dynamic process in which both newly formed oligodendrocytes and pre-existing oligodendrocytes remodel myelin, often in response to activity, to facilitate learning and plasticity [35]. Whether activity-dependent remyelination could be restored by the proposed treatments remains an unanswered question.

## Biology of remyelination

Demyelination (induced experimentally or by disease) can be followed by this regenerative response leading to the formation of new myelin sheaths around denuded axons by newly formed oligodendrocytes [23, 36-39]. Histopathological assessments have highlighted that this can occur extensively in some people with MS [40], but is inadequate in a significant proportion [41, 42]. For example, one study analysed forebrain tissue from 51 MS patients and found widespread remyelination in 20% of individuals, yet 34 cases remyelinated fewer than 25% of their lesions [42]. High inter-subject variability in remyelination capacity is supported by dynamic myelin imaging using positron emission tomography (PET) [43] and, when combined with evidence that those demonstrating more remyelination display lower levels of disability [44], it underscores the therapeutic promise of a remyelinating treatment. Consequently, efforts have been made to understand the mechanisms of remyelination and why this fails in MS, in the hope of defining druggable targets to enhance this process.

### Mechanisms of remyelination

While pre-existing mature oligodendrocytes are able to increase the number of internodes they generate, and therefore contribute to recovery after demyelination [45], they do not add to the pool of new myelinogenic oligodendrocytes that are required for remyelination in animals [46]. Thus, remyelination is crucially dependent upon adult oligodendrocyte progenitor cells (aOPCs), derived from neonatal OPCs (nOPCs) [47], which have been shown by genetic fate-mapping to be the cells responsible for generation of the majority of new oligodendrocytes in the adult nervous system [48, 49]. These cells are maintained in sufficient quantities predominantly by their own self-renewal, rather than by replacement from neural stem cell niches in the CNS [50, 51].

Following damage to myelinated areas, aOPCs must follow a choreographed process of activation, migration, proliferation and differentiation before culminating in the formation of new myelin sheaths [48]. The final product is a compacted layer of myelin that is thinner and shorter than those formed during developmental myelination [52], a fact often used to identify remyelination histologically when the process is studied in animal models (Box 1). Mechanistically, remyelination might fail due to a defect anywhere in this sequence; a paucity of pro-regenerative factors, or excess of inhibitory factors, as can be seen in MS lesions, combined with the intrinsic composition of the aOPC, can limit the capacity to remyelinate [23].

**Box 1:** Animal models used to study remyelination

Remyelination has been studied in several animal models:
Experimental autoimmune encephalomyelitis (EAE): this model of autoimmune inflammation, driven by injection of a myelin peptide alongside an adjuvant, sees inflammation and remyelination occurring concurrently. However, when used experimentally to explore potential medicines, it is often hard to distinguish an effect of attenuation of inflammation from promotion of remyelination. Hence non-inflammatory models have been developed, as below
Gliotoxin injections: lysolecithin and ethidium bromide (EB) are toxic to oligodendrocytes, yet spare axons. Experimentally, they can be injected into the CNS of animals to induce demyelination. Their particular benefit has been that the kinetics of demyelination and remyelination can be closely studied [162]. The limitation is that the lesions do not necessarily model the complexity of those in multiple sclerosis, which contain a myriad of remyelination inhibitors and inflammatory cells
Oral cuprizone administration: dietary ingestion of the copper chelator cuprizone results in demyelination of white matter tracts, particularly in the corpus callosum [163]. It models remyelination, ongoing in the face of continued demyelination. However, the normally small diameter axons seen in the corpus callosum makes distinguishing a remyelinated from an unaffected axon challenging, and interpretation correspondingly difficult
The best model for progressive MS is debated and variations of these employed (reviewed in [164]). Our view is, for reasons that will become apparent, that experiments should be performed in aged animals when studying the underlying mechanisms of remyelination failure in progressive MS

Given that large numbers of aOPCs are seen in chronically demyelinated MS lesions [53], it is often stated that remyelination fails as aOPCs become quiescent and unable to differentiate. As a consequence, increasing research has been deployed to elucidate the key regulators of differentiation [54-58] and identify agents capable of enhancing this process for clinical use, which is discussed further below.

However, it should be acknowledged that differentiation may not universally be the rate-limiting step in humans. It has previously been established that aOPCs do migrate to sites of injury and evenly distribute themselves to facilitate remyelination [59], though they probably do so over short distances [60]. But recently, two papers have also raised questions about the contributions of aOPCs to lesion repair in people. Yeung and colleagues utilised a ^14^C dating technique to show that oligodendrocytes from shadow plaques in MS brains, areas thought to have undergone at least partial remyelination, could not have been generated from new OPCs [61]. Further, Jäkle et al. reported that at autopsy, OPCs are reduced in number within shadow plaques [62]. Indeed, this latter study also highlighted changes in oligodendrocyte gene expression profiles between areas of normal-appearing white matter of MS brains and healthy controls, implying that the pathology seen in lesions may not reflect the global cellular changes occurring in MS.

Such results may find explanations in the differences between humans and the rat and mouse models used to study the disease. Taken together with evidence that some MS patients remyelinate better than others [42, 43], it seems clear that a treatment strategy that enhances differentiation alone may not necessarily be sufficient to address remyelination across a population of heterogenous MS patients.

Further, interactions between other glial cells and OPCs are also being increasingly clearly defined. Reactive astrocytes found at the site of demyelination, for example, secrete inhibitors of remyelination such as Endothelin-1 [63] and the recent description of A1 reactive astrocytes, which contribute to the death of oligodendrocytes [64], needs to be incorporated into the current model of remyelination and potential therapeutic targets explored.

In parallel, the demonstration that protein synthesis in OPCs is modulated by axonal action potentials [65] speaks to an underlying symbiosis between the neuron and the cells responsible for its myelination. In the peripheral nervous system, there is a necessary relationship between axon and Schwann cell, exemplified by dependency on the neuron-derived growth factor Neuregulin 1 to drive peripheral nerve myelination [66]. In the CNS, OPCs are able to differentiate even in the absence of axons [67, 68] and, as will be discussed below, in culture are able to myelinate inert axon-like substrates [69, 70]. Yet, while oligodendrocytes have a default ability to differentiate and myelinate axons, this is modulated by axon diameter and activity, implying a requirement for intact axons in vivo [71].

Therefore, it looks increasingly probable that combinations of drugs, acting on different processes, will be required to facilitate remyelination, and that these will be most effective when there is a sufficiently preserved demyelinated axon. This latter point forms the rationale for many phase 2 studies first focussing on people with RRMS, in whom it is anticipated that fewer axons will have degenerated.

### Reasons for remyelination failure

To understand why remyelination fails in MS, one must look at two crucial contributory processes—namely those of age and the immune system.

While the immune system is often seen as having a detrimental role in MS, the innate immune system has been shown to be essential in the biology of remyelination [72]. Myelin debris contains inhibitors of aOPC differentiation and so its clearance, by phagocytosis, is an important step in the regeneration of the myelin sheath [73-76]. Similarly, infiltrating macrophages and activated microglia secrete a myriad of neurotrophic factors, which have direct effects on aOPCs [77]. Indeed, to facilitate robust remyelination in vitro, the polarisation of the macrophage response to an immunoregulatory, “M2”, phenotype is required [78]. It is not clear how these findings relate to the behaviour of monocyte-derived macrophages and microglia, in vivo, yet they emphasise how improving our understanding of subpopulations of macrophages/microglia and lymphocytes in the brain is essential to developing treatments that prevent demyelination while promoting remyelination.

The potential for endogenous remyelination is both age and disease duration dependent: remyelination is greatest in people aged less than 55 years and within the first 10 years of disease onset [39, 79, 80]. Disentangling the relative contribution of age versus duration of lesion demyelination to remyelination failure remains to be done, but clinical evidence would suggest age is especially pertinent as patients reach disability milestones at similar ages whether they have relapsing or progressive symptoms at onset [8, 80]. Similarly, lesional magnetisation transfer ratio (MTR)—a putative marker of remyelination—also shows age-dependent decline [81]. Remyelination is therefore akin to other regenerative processes [82] in becoming less efficient with time [83-87]; understanding age-associated remyelination failure is essential in treatment development.

Mechanistically, in this circumstance, the rate-limiting step is more clearly differentiation of the aOPC, as increasing aOPC recruitment does not lead to enhanced remyelination in aged mice [88]. Studies of how extrinsic factors vary with age have implicated a declining efficiency of the inflammatory response [89]; as noted above, macrophages produce pro-differentiation factors and clear debris by phagocytosis [76, 90, 91], which is essential for remyelination. That this process might be modifiable was demonstrated by the reversal of a deficit in remyelination of an aged mouse by twinning its circulation with a young animal by heterochronic parabiosis [92]. In a similar way, small molecule treatments can be used to promote endogenous remyelination, even in aged animals.

A detailed understanding of the intrinsic age-related changes in aOPCs is a recent development in the field following work by Neumann and colleagues [93]. They demonstrated that aged aOPCs become less responsive to factors that induce differentiation, contributing to the reduced remyelination capacity seen in many non-remyelinating chronic MS lesions [94]. Moreover, RNA sequencing from young and aged aOPCs highlighted a significant contribution from the mTOR pathway. This led to the novel observation that manipulating this pathway in aged rats with caloric restriction (three non-consecutive days of fasting per week over 6 months), or with the AMPK-agonist metformin (over 3 months), reverses the diminished differentiation capacity of aOPCs and restores their ability to remyelinate. As a result, manipulation of intrinsic changes in these stem cells is emerging as a promising treatment strategy.

Finally, there are also anatomical variations to the extent of remyelination within different lesions in the same individual. For example, periventricular lesions are less amenable to remyelination than subcortical lesions [42, 80]. This might reflect an underlying heterogeneity in OPCs or in locational differences in permissibility for their differentiation [95]: there are fewer inhibitors of remyelination in the cortex [96]. It could also be due to the importance of neuronal activity for remyelination, which is more likely to occur closer to the soma. Regional variations in remyelination within an individual is an opportunity to investigate barriers to enhancing remyelination, but also raise questions about which lesions should be tested in clinical trials.

## Identification of agents capable of remyelination

An enhanced understanding of the intrinsic and extrinsic regulatory pathways implicated in remyelination has identified a multitude of sensible targets for therapeutic manipulation. An example of this has been the development of opicinumab to inhibit Lingo-1 (leucine-rich repeat and immunoglobulin-like domain-containing nogo receptor-interacting protein 1), a negative regulator of differentiation [97].

Another fruitful technique has been high-throughput screening of libraries of compounds, looking for an effect on aOPC differentiation [98]. One such study focussed on the ability of candidate compounds to promote differentiation of rat optic nerve-derived progenitor cells as evidenced by their production of oligodendrocyte differentiation markers [99]. This revealed that antagonism of muscarinic receptors, with the antihistamine/anticholinergic benzatropine, promotes OPC differentiation in vitro, which translated into a remyelinating effect in both EAE and cuprizone mice models. Similarly, Najm et al. used a flat plate culture system to screen a library of bioactive small molecules, this time on mouse pluripotent epiblast stem cell-derived OPCs. They discovered that the topical corticosteroid, clobetasol, and the anti-fungal, miconazole, as well as benzatropine, lead to a mature oligodendrocyte morphology, and improved remyelination in a lysolecithin-induced mouse model of focal demyelination [100].

A slightly different approach has used concentric wrapping of myelin around micropillars as an end point rather than differentiation per se. Mei et al. assessed the ability of 1000 FDA-approved small molecules to promote OPCs and oligodendrocytes to ensheath these cone-like structures with myelin [69]. In this way, they identified a cluster of compounds with an anti-muscarinic effect: atropine, ipratropium, oxybutynin, trospium, quetiapine, benztropine and clemastine. This work was quickly translated into the positive phase 2 trial of clemastine as a remyelinating therapy [101], discussed below.

Such small molecules may not have their remyelinating effect through an exclusive action at their canonical targets. The closest to a unifying mechanism has been through demonstration that a wide range of these, including clemastine, benztropine, miconazole and ketoconazole, might promote remyelination through altering the sterol landscape in the OPC to favour accumulation of 8,9-unsaturated sterols [102].

However, these techniques predominantly rely on the assumption that OPC differentiation is the rate-limiting step in remyelination, which might not be as rational as once thought [61, 62, 103]. The micropillar array is also limited in its ability to test the development of functional architecture in the form of nodes and internodes. It follows that combination therapies may be necessary to optimise an effect across the population of MS lesions. Moreover, such efforts will inevitably be hampered by the lack of an animal model that encapsulates the complexity of the MS lesion; there is a risk that agents showing promise in preclinical work do not translate into a beneficial effect in humans or indeed that a potentially useful treatment effect is missed in such models, halting progression towards clinical studies [23].

## Remyelination clinical trials

The identification of agents that therapeutically enhance endogenous remyelination in preclinical models has led several to be translated into clinical trials and the possibility of a neuroprotective treatment in MS looks increasingly likely. In Table 1, we summarise the clinical trials that have been performed while considering a few in more detail below.

**Table 1:** Clinical trials of remyelination treatments in multiple sclerosis

Treatment (trial name, NCT ID)	Information	Primary outcome	Status/result (references)
Liothyronine (MST3K, NCT02760056)	A phase I, randomised, double-blind, placebo-controlled, dose-finding trial of liothyronine sodium (L-T3) given for 1 week to people with any type of MS	Maximum tolerated dose of L-T3 as measured by hyperthyroid symptom scale	Completed. Results awaited
Interferon β1a/rebif (NCT01085318)	A phase IV, open label study of Rebif 44 mcg three times weekly in people with RRMS compared to healthy controls	Change in volume of normal-appearing brain tissue (NABT) showing increasing MTR at 24 weeks	Completed. Failed to meet primary end point, though NABT volume with increasing MTR was greater in patients at 12 weeks compared to healthy controls [165]
Natalizumab/tysabri (NCT00937677)	A phase IV, open label, observational, single-blinded study of people with relapsing and relapsing progressive MS. 77 treated with natalizumab, 26 treated with intramuscular interferon, 22 healthy controls	Change in volume of NABT with increasing MTR at 2 years	Volume of NABT undergoing increases in MTR higher in natalizumab-treated group compared to interferon and healthy controls [166]
CNM-Au8 Nanocrystalline gold (NCT03536559)	A phase II, randomised, double-blind, placebo-controlled, parallel group study in 150 people with MS and evidence of chronic optic neuropathy	Multifocal visual evoked potential latency at 24 weeks	Recruiting
Bexarotene (CCMR One)	A phase II, randomised, double-blind, placebo-controlled trial of 50 people with RRMS treated with dimethyl fumarate	Change in mean lesional MTR in chronic lesions with an MTR below the within-patient median	Completed. Results awaited
Alemtuzumab (NCT01395316)	A phase IV cohort study, in group of 8 patients with MS treated with two cycles of alemtuzumab	Diffusion and MWF changes on MRI	Completed. Results awaited
Adrenocorticotrophin, ACTH (NCT02446886)	A phase IV, randomised, open-label study of ACTH gel on remyelination in patients with RRMS or SPMS and new contrast-enhancing lesions	Change in MWF within new Gd-enhancing lesions over the course of 12 months	Recruiting
GSK239512 (NCT01772199)	A phase II, randomised, placebo-controlled, single-blind study in 131 people with RRMS on interferon or glatiramer acetate	Mean change in Gd-enhancing lesion MTR from before enhancement to stable recovery	Completed. Small positive effect observed in treated group [114]
Biotin/MD1003 (MS-SPI, NCT02220933)	A phase III, randomised, placebo-controlled, double-blind trial of high-dose biotin in 154 people with SPMS or PPMS	Disability reversal with EDSS decrease of > 1 or > 20% decrease in T25FW	Completed. 12.6% of treated patients achieved primary end point versus none of the untreated patients [120]
Biotin/MD1003 (MS-SPII, NCT02936037)	A phase III, randomised, placebo-controlled, double-blind trial of high-dose biotin in 642 people with SPMS or PPMS	As above	Ongoing
Biotin/MD1003 (MS-ON, NCT02220244)	A phase III, randomised, placebo-controlled, double-blind trial of high-dose biotin in 93 people with SPMS or PPMS	Change in visual acuity over 6 months	Completed. No significant changes in visual acuity [121]
Olesoxime (MSREPAIR, NCT01808885)	A phase Ib, randomised, placebo-controlled, double-blind trial of olesoxime compared to placebo in people with RRMS	Safety criteria, though MTR included in exploratory outcome measures	Completed
Clemastine (ReBUILD, NCT02040298)	A phase II, randomised, placebo-controlled, double-blind, crossover trial in 50 people with RRMS and chronic stable optic neuropathy	Change in P100 latency of the full-field VEP	Completed. Significant latency reduction of 1.7 ms in the crossover model and 3.2 ms in delayed treatment analysis [101]
Clemastine (ReCOVER, NCT02521311)	A phase II, randomised, double-blind, placebo-controlled trial in 90 people diagnosed with acute demyelinating optic neuritis	Change in P100 latency of the full-field VEP and change in low contrast visual acuity	Recruiting
Quetiapine (NCT02087631)	A phase I/II open label, dose-ranging study of quetiapine in people with RRMS and PMS	Dose-limiting toxicity, no specific remyelination outcomes	Recruiting
Opicinumab (RENEW, NCT01721161)	A phase II, randomised, placebo-controlled, double-blind, study of opicinumab in subjects with a first episode of acute optic neuritis	Change in VEP P100 latency in affected eye, referenced to the unaffected eye, over 24 weeks of treatment	Completed. Significant improvement in latency, but only on per protocol analysis [109]
Opicinumab (SYNERGY, NCT01864148)	A phase II, randomised, placebo-controlled, double-blind trial of opicinumab (at 3, 10, 30, or 100 mg/kg) in 418 subjects with RRMS treated with interferon β1a	Change in performance at EDSS, T25FW, 9HPT and 3 s-PASAT	Completed. Did not meet primary end point, but increased percentage of improvement responders at 10 and 30 mg/kg doses [110]
Opicinumab (AFFINITY, NCT03222973)	A phase II randomised, double-blind, placebo-controlled study of opicinumab versus placebo in 263 people with RRMS	Overall response score composed of EDSS, T25FW and 9HPT from each hand	Ongoing
rHIgM22 (NCT02398461)	A phase I randomised, double-blind, placebo-controlled study of rHIgM22 compared to placebo in 27 people with RRMS following a relapse	Safety and tolerability end points	Completed [167]
Domperidone (NCT02493049)	A phase II randomised, open-label, single-blind study of domperidone 10 mg three times daily in people with RRMS and new Gd-enhancing lesions	Texture analysis, DTI and MTR in enhancing lesions over 32 weeks	Ongoing

*NABT* normal appearing brain tissue, *MTR* magnetization transfer ratio, *Gd* gadolinium, *MWF* myelin water fraction, *DTI* diffusion tensor imaging, *RRMS* relapsing remitting multiple sclerosis, *SPMS* secondary progressive multiple sclerosis, *PMS* progressive multiple sclerosis, *IFN* interferon, *VEP* visual evoked potential, *EDSS* Expanded Disability Status Scale, *T25FW* timed 25-foot walk, *9HPT* 9-hole peg test, *PASAT* paced auditory serial addition task

### Clemastine

This is a first generation anti-histamine that was identified in the micropillar array as being capable of stimulating OPCs to differentiate and carry out the first stages of myelination [69]. This was confirmed in a further screen [99] and shown to occur via an off-target anti-muscarinic action, likely a specific effect on the M1 muscarinic receptor [29]. Ensuing work would confirm its remyelinating effect in multiple animal models [29, 69, 104, 105].

As clemastine has been licensed for allergic rhinitis since 1992, it was readily translated into a clinical trial [101]. The ReBUILD study was a single-centre, double-blind, randomised, placebo-controlled, phase 2, crossover trial, which specifically investigated the remyelinating potential of clemastine in patients with RRMS and evidence of chronic demyelinating optic neuropathy. Their inclusion criteria ensured that there was detectable demyelination in the optic pathway (evidenced by a visual evoked potential (VEP) P100 latency > 118 ms in at least one eye), but also sufficient axons to regenerate [with a retinal nerve fibre layer thickness (RNFL) > 70 μm in the qualifying eye when measured with optical coherence tomography (OCT)]. Meanwhile, the remyelination that might be expected in the natural history of optic neuritis was excluded by selecting only those without a history of acute optic neuritis in the qualifying eye within the last 5 years, or in either eye in the last 6 months.

The study design saw participants divided into two groups, but ensured that all had access to the study drug (which is readily available in the USA without prescription). 25 were given 5.36 mg of clemastine twice daily for 90 days followed by placebo for 60 days (group 1), while a further 25 patients were given placebo for 90 days followed by clemastine for 60 days (group 2).

The results of this were rather promising. VEP P100 full-field latency was reduced by 1.7 ms/eye (*p* = 0.0048) in the crossover model. Furthermore, the clinical effect of clemastine was sustained in group 1 after switching to placebo. Thus, the crossover model underestimates the actual effect, later demonstrated to be a 3.2 ms reduction in P100 latency. Further, there was a significant improvement in a functional outcome, low contrast letter acuity, when the delayed treatment analysis was employed. All the while, the drug was well tolerated, though was associated with fatigue. Secondary end points were negative, however, including MRI assessments of myelin water fraction (MWF), whole brain MTR, white matter MTR, and white matter fractional anisotropy (FA). There was no effect on the Expanded Disability Status Scale (EDSS), a timed 25-foot walk and the 6-min walk test.

This positive trial has provided some optimism about clemastine, though its selective inclusion criteria raise the possibility that the results are not generalisable; 75 patients were excluded based on VEPs. The ReCOVER trial (NCT02521311) will, no doubt, advance things further as it investigates the effect of clemastine (4 mg three times daily for 1 week, followed by 4 mg twice daily until 3 months treatment) in patients with acute optic neuritis. However, its clinical role needs further definition before widespread use: the ReBUILD result requires progression to phase 3 studies, clemastine should be trialled in progressive cohorts, and the possibility of combining treatments, such as with the potential synergistic effect of metformin, requires investigation.

### Opicinimab

As mentioned above, Lingo-1 is a negative regulator of oligodendrocyte differentiation and its antagonism has been shown in vitro and in animal models of CNS demyelination to enhance remyelination [106]. The human monoclonal antibody opicinumab (anti-Lingo-1) showed remyelinating activity in preclinical studies [107] and therefore its utility was explored in early clinical trials. After passing safety analyses in a phase 1 trial [108], there was a phase 2, randomised, double-blind, placebo-controlled, clinical trial (RENEW) in patients with a first episode of acute optic neuritis (but not necessarily multiple sclerosis) [109]. The primary outcome measure was the recovery in VEP P100 latency in the affected eye, referenced to the unaffected eye, over 24 weeks of treatment (at 100 mg/kg) after an episode of optic neuritis. It failed in this regard but, when a per-protocol analysis was employed, an improvement of 7.6 ms in the opicinimab group was seen over that in the placebo group. No change was observed in the secondary end points, though this did not include MRI sequences such as MTR.

The SYNERGY trial followed (NCT01864148): a dose-ranging study including 418 people with RRMS and SPMS. The primary outcome measure was a composite of ambulation (25-foot walk), upper extremity function (9-Hole Peg Test), cognition (3-Second Paced Auditory Serial Addition Test; PASAT) and the EDSS. It failed to meet this primary end point, but was presented as showing an increased percentage of responders in those treated with the mid-range doses of 10 and 30 mg/kg [110]. This has led to Biogen proceeding with a refined phase II trial (AFFINITY) in addition to an extension study (RENEWED, NCT02657915) of the RENEW trial.

### GSK239512

This H3-receptor antagonist was originally developed to treat Alzheimer’s disease [111, 112] and was put forward as a potential remyelinating agent because H3 negatively regulates oligodendrocyte differentiation [113]. A phase 2, randomised, placebo-controlled study in people with RRMS on interferon or glatiramer acetate revealed a small, but significant, improvement in mean change in post-lesion MTR in gadolinium-enhanced lesions compared to placebo [114].

### Bexarotene

Robin Franklin’s group demonstrated that, in aged animals, 9-cis-retinoic acid enhances remyelination through agonism of the nuclear retinoid acid receptor RXR-γ [115]. As a similar effect could be achieved by a pan-RXR agonist licensed to treat skin lymphoma, bexarotene [116], this facilitated translation into a phase 2 clinical trial (CCMR One). Indeed, while an RXR-γ specific agonist might be more desirable, an effect of RXR-α activity on remyelination was also later reported [117].

The CCMR One study utilised MTR to quantify remyelination as a primary measure. However, in contrast to other studies, its focus is on changes in mean lesional MTR between month zero and month six for the lesions selected, for each patient, whose MTR lies below the within-patient median. In this way, it is hoped that the outcome will focus on an effect on lesions that are demyelinated at the baseline visit [118].

### Biotin

Biotin is postulated to promote remyelination when given in high doses through its role as a cofactor for carboxylases required for fatty acid synthesis in oligodendrocytes [119]. To date, clinical trials have focussed on cohorts of progressive patients. The MS-SPI study showed a small statistically significant effect in 12.6% of treated participants when using an improvement of either the EDSS or timed 25-foot walk as its outcome [120]. However, the MS-ON study returned a negative result when change in visual acuity was employed as the primary end point [121]. It therefore remains uncertain whether high-dose biotin could be a clinically useful treatment for people with MS—and is under further investigation in MS-SPII—yet these trials also highlight the importance of selection of patient group and outcome measures, which will be discussed further below.

### Cell-based therapies

Enhancing the activity of endogenous oligodendrocyte progenitors has proved the most accessible strategy to promote remyelination to date, therefore forming the focus of this review. However, other non-ablative cell-based approaches have been generating substantial interest (reviewed in [122]): transplantation of mesenchymal stem cells (MSCs), derived from bone marrow or other tissues, and transplantation of OPCs, derived from foetal tissue, embryonic stem cells or induced pluripotent stem cells (iPSCs) [123, 124] are viable options, but remain experimental. Challenges exist for each with regard to cell production, mode of delivery, tumour-forming potential, and requirements for immune suppression, although using autologous sources may abrogate the need for the latter. One noteworthy, albeit uncontrolled, trial administered bone marrow-derived MSCs to ten patients with progressive MS, noting an improvement in VEP latency of 1.3 ms, interpreting the mechanism for this as a neuroprotective effect through the promotion of myelin repair [125]. Larger phase 2 studies are underway [126], though there are many unresolved barriers to widespread application of a transplant-based approach to MS.

## The target population for remyelination therapies

The efficacy of a remyelinating therapy would be greatest early in the course of MS, to stop long-term axonal degeneration and so prevent, or at least slow the onset of, secondary progression. More problematic is how effective such a therapy would be later in the disease course, for instance in progressive multiple sclerosis, when presumably many axons have already degenerated and so there is no scaffold for remyelination. Ultimately, this question can only be resolved by clinical trials. For the moment, we would advocate testing potential remyelinating agents in trials of patients identified as having demyelinated lesions with axons still present, such as those in the ReBUILD trial.

## Determining remyelination in clinical trials

One of the foremost challenges to translating promising preclinical findings into clinical studies is uncertainty in the optimum way to demonstrate a remyelinating effect in living individuals. Given that the anticipated benefit to the patient, the prevention or delay to progression, only manifests over years, reliance on standard objective clinical markers of disability, for example the EDSS [127], or indeed on functional measures such as visual acuity, as outcomes may miss a useful therapeutic effect over a comparatively short clinical trial. Moreover, using functional scores to study remyelination specifically is further complicated by other adaptations that occur in nerves in response to injury, such as ion channel redistribution and cortical plasticity/adaptation after demyelination [128, 129]. There follows a reliance on paraclinical measures to determine a treatment effect; with no biomarker of myelin regeneration in biological fluids and the lack of accessible tissue for histological examination, a combination of neurophysiological and imaging-based assessments are required [130].

### Neuroimaging

From the imaging perspective, standard MRI measures (such as T2-weighted and gadolinium-enhanced T1-weighted sequences) correlate only modestly with disability and lack the ability to differentiate between pathological correlates of MS: namely inflammation, oedema, axonal loss, demyelination, remyelination and gliosis [131]. As a result, advanced MRI techniques including myelin water fraction (MWF) [132, 133], diffusion tensor imaging (DTI) [134, 135] and magnetisation transfer ratio (MTR)[136, 137], as well as positron emission tomography (PET) [43, 138], have been variably used to measure myelin dynamics.

Magnetisation transfer techniques investigate the exchange of magnetisation between protons in at least two pools: those that are mobile and those associated with macromolecules such as myelin or axonal membranes. Expressed as a ratio (MTR), it provides a quantitative measure of the proportion of protons bound to macromolecular structures relative to those that are free in water and has been demonstrated to correlate with pathological quantification of myelin: demyelinated lesions have a significantly lower MTR than remyelinated lesions [137]. MTR can be used to quantify myelin in several ways. One can use serial measures of mean MTR in white and grey matter [139], in chronic lesions [118] or in acute (gadolinium-enhancing) lesions [81]. Indeed, as not all lesions remyelinate to the same extent, further refinements have been proposed. For example, in the CCMR One study of bexarotene, the primary outcome measure is based on the change in mean MTR of established lesions with a low MTR at the baseline scan, thereby maximising sensitivity to an effect on lesions that are demyelinated at the outset [118]. These aforementioned techniques have been used to show that mean MTR in white and grey matter remains static over time in people with MS treated with alemtuzumab, but deteriorates if not on disease-modifying treatment [139], and that the anti-histamine GSK239512 has a small positive effect on mean MTR in gadolinium-enhancing lesions [114].

Alternatively, DTI provides information about tissue microstructure by measuring water diffusion in vivo. Parameters derived from this such as radial diffusivity (a marker of water motion perpendicular to the axon) as well as the overall fractional anisotropy can be used as a surrogate of myelin content [135, 140]. Meanwhile, MWF indicates the proportion water trapped between myelin bilayers relative to water inside and outside of axons (which have different T1 and T2 relaxation times), and can be used as a proxy for myelin content [132, 141].

Finally PET imaging, alongside a myelin-specific ligand such as Pittsburgh compound B (PiB), might be especially sensitive to changes in myelin [142] and could potentially be used to stratify the patients by their remyelination potential for clinical studies [43]. However, the issues of availability and radiation exposure are likely to limit the role this has to play.

### Neurophysiology

Evoked potentials allow for an assessment of nervous conduction along visual, somatosensory, auditory, and motor tracts in a way that correlates with function [143-145] and disability [146], but their clinical utility, particularly in diagnostics, has largely been replaced by MRI [147]. However, such indices are proving invaluable as biomarkers in assessing remyelination; in the recent positive phase 2 trial of clemastine, it was a reduction in VEP P100 latency, rather than clinical or imaging markers, that confirmed the biological effect [101, 148].

The pattern reversal VEP represents the average recordable electric potential in the visual cortex in response to the presentation of an alternating checkerboard-patterned stimulus. The main focus is on the positive deflection in the VEP waveform approximately 100 ms after the visual stimulus (the P100), which provides measures in the form of latency (a surrogate of demyelination in the optic pathways) and amplitude (predominantly a function of axonal loss). Following an attack of optic neuritis, VEP latencies are prolonged but a period of recovery follows, most significantly within the first 6 months, but for perhaps as long as 3 years [149, 150]. Meanwhile, in those with chronic stable optic neuropathy, a prolonged P100 latency is seen, which has been shown in longitudinal data to remain stable, or gradually lengthen, with time [151]. As a result, in studies of patients without a recent bout of optic neuritis, improvements in VEP P100 latency can be used as a marker of remyelination; this was the rationale behind the ReBUILD trial [101]. When studies have enrolled patients with acute optic neuritis meanwhile, such as in the RENEW study of opicinumab [109], values for the unaffected contralateral eye have been used as a control and the outcome measure given as the change in latency difference between the two eyes.

The clinical heterogeneity of MS has already been alluded to, which extends beyond a consideration of optic neuritis. Given the emerging importance of neurophysiological measures in such studies, a more robust biomarker in future trials might be a combination of VEPs, motor EPs (MEPs), somatosensory EPs (SEPs), and brainstem auditory EPs (BAEPs) [152]. Such “multimodal” evoked potentials can be combined to give a “global” outcome, which has previously been shown to correlate with disability and inform disease progression [153], and have already been employed in the field of bone marrow-derived cell therapy [126, 154]. Indeed other exploratory, neurophysiological techniques have also been emerging, such as the use of saccadometry [155], most recently showing improved conduction along the medial longitudinal fasciculus in the setting of internuclear ophthalmoparesis treated with fampridine [156].

### Other techniques

Optical coherence tomography (OCT) is another tool being employed to indirectly assess remyelinating therapies. By generating high-resolution images of the retina to gauge axonal loss in the retinal nerve fibre layer (RNFL) [157], which is decreased following optic neuritis [158], it provides a measure of neurodegeneration rather than specifically remyelination, but can be used to complement the analysis of the VEP [159]. Similarly, serum neurofilament, a marker of axonal damage that correlates with MS disease activity [160], has been postulated to be a valuable outcome measure for remyelination trials. A subset of participants from the SYNERGY trial (discussed above) showed a trend toward neurofilament light decline among treatment responders [161]; however such measures remain an indirect marker of remyelination and, in our view, should remain exploratory.

## Conclusions

People living with multiple sclerosis in resource-rich regions now have access to a range of anti-inflammatory treatments which promise long-term disease modification if given early in the course of the relapsing–remitting phase of the disease. However, even the most effective of these treatments leaves significant numbers of axons demyelinated and vulnerable to degeneration, which is the substrate of progressive disability. Finding remyelinating therapies, with the potential to both restore function and prevent axon degeneration is therefore an urgent clinical need.

A host of strategies have been identified from preclinical research, and some have now been translated to early phase clinical trials. Two of these have yielded positive results on surrogate measures, such as VEP latency or lesional MTR, whose clinical validity is yet to be demonstrated. There have also been setbacks, such as the failure of anti-Lingo-1 antibodies in phase 2 trials to replicate their in vitro efficacy.

In this review, we have highlighted a number of unresolved questions the scientific community faces. First, to ensure we take the right drugs forward, we must continue to develop an understanding of the barriers to remyelination, specifically in humans, and better appreciate how biology observed in animal models of remyelination translates into human disease. Second, we need to identify which is the best, most reliable, measure of remyelination to use in clinical trials. Third, we must clarify when is the best time to initiate a remyelinating treatment in the disease course. Finally, we need to consider which is the most appropriate test population for our trials. Ultimately, more work is required to reach a reality of neurologists prescribing neuroprotective treatments to people with MS; however, advances in the last decade have made this look increasingly probable.
